# Experimental obstructive cholestasis: the wound-like inflammatory liver response

**DOI:** 10.1186/1755-1536-1-6

**Published:** 2008-11-03

**Authors:** María-Angeles Aller, Jorge-Luis Arias, Jose García-Domínguez, Jose-Ignacio Arias, Manuel Durán, Jaime Arias

**Affiliations:** 1 Department of Surgery I, School of Medicine, Complutense University of Madrid, Spain; 2 Neurosciences Laboratory, School of Psychology, University of Oviedo, Asturias, Spain; 3 Plastic and Reconstructive Surgery Unit, Getafe Hospital, Madrid, Spain; 4 General Surgery Unit, Monte Naranco Hospital, Oviedo, Consejería de Salud y Servicios Sanitarios, Principado de Asturias, Spain; 5 Health Sciences School, King Juan Carlos University, Madrid, Spain

## Abstract

Obstructive cholestasis causes hepatic cirrhosis and portal hypertension. The pathophysiological mechanisms involved in the development of liver disease are multiple and linked. We propose grouping these mechanisms according to the three phenotypes mainly expressed in the interstitial space in order to integrate them.

Experimental extrahepatic cholestasis is the model most frequently used to study obstructive cholestasis. The early liver interstitial alterations described in these experimental models would produce an ischemia/reperfusion phenotype with oxidative and nitrosative stress. Then, the hyperexpression of a leukocytic phenotype, in which Kupffer cells and neutrophils participate, would induce enzymatic stress. And finally, an angiogenic phenotype, responsible for peribiliary plexus development with sinusoidal arterialization, occurs. In addition, an intense cholangiocyte proliferation, which acquires neuroendocrine abilities, stands out. This histopathological finding is also associated with fibrosis.

It is proposed that the sequence of these inflammatory phenotypes, perhaps with a trophic meaning, ultimately produces a benign tumoral biliary process – although it poses severe hepatocytic insufficiency. Moreover, the persistence of this benign tumor disease would induce a higher degree of dedifferentiation and autonomy and, therefore, its malign degeneration.

## Background

Obstructive cholestasis is characterized clinically by jaundice, discolored urine, pale stools and pruritus [1-3]. Obstruction of the biliary tree, either intrahepatic or extrahepatic, induces a characteristic pattern of early and late liver morphologic features that can be attributed to the evolution of an inflammatory response [3].

Hepatic inflammation is an important feature of cholestasis liver disease in both humans [1] and experimental animals [4,5]. Inflammatory features of obstructive cholestasis include portal tract edema [3], neutrophil infiltration in the portal tracts [3,5], proliferation of the biliary epithelial cells [1,3] and portal tract fibrosis [1,3,6].

The preferable localization of the inflammatory response in the portal tract reflects the importance that this space occupies in the development of the hepatic cholestasis pathology. In this way, the main role of the portal tract in hepatic inflammation is similar to that of the interstitial space during inflammation of other organs or tissues [7,8]. This is why both spaces, the portal tract and the interstitium, can be considered similar to a certain extent.

## The functional biliary tree in the interstitial space

The biliary tree begins with the 'source of the bile', which is the bile canaliculus, made up by the canalicular domain of adjacent hepatocytes [3]. Bile canaliculi form a network of channels between hepatocytes and drain into the canals of Hering (intrahepatic bile ducts) that are lined by hepatocytes and cholangiocytes and also contain hepatic stem cells [3,9-11].

**Figure 1:**
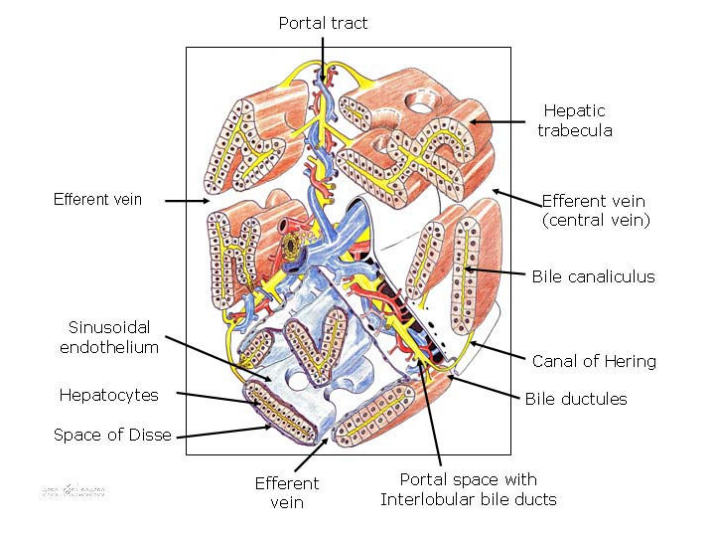
**Schematic 3D representation of a complex acinus according to Rappaport**. Three portal tracts diverge from one axis, made up of the bile duct, the hepatic arterial and portal venous branches. At the same time, several trabeculae or laminae hepatis that are two cells thick arise from these three portal spaces and are oriented towards the efferent veins (central veins). In the lower part of the drawing, the liver plates are covered by the sinusoidal endothelium and the space of Disse located between both structures contains tissue fluid, which flows outwards into the lymphatics of the portal zones. The space of Disse continues with the portal space, and they both make up the interstitial space of the acinus.

The canals of Hering continue into the bile ductules, which in turn drain into interlobular bile ducts, located in the portal space (Figure 1). Bile ductules and interlobular bile ducts are composed entirely of cholangiocytes. Interlobular bile ducts then continue into progressively larger ducts and finally drain into the extrahepatic biliary tract. The extrahepatic biliary tract is grossly divided into the common hepatic duct, the common bile duct, the cystic duct and the gall bladder [3,9,11]. The intrahepatic and extrahepatic biliary tract are supplied and nourished by a network of fine vessels called the peribiliary vascular (or capillary) plexus, derived from the hepatic artery [3,9]. The liver has a connective envelope, that inflexes at the level of the hilum and follows ducts and vessels within the organ until the periportal spaces. The periportal spaces represent the center of the functional units of the organ. From the portal tract, the blood supplies feed into vascular sinusoids that are bounded by laminae of hepatocytes that finally drain into the efferent central vein [12].

Between the sinusoidal endothelium and the vascular pole of the hepatocytes lies the space of Disse (perisinusoidal space) [12] (Figure 1). This space contains the extracellular matrix (ECM) and hepatic stellate cells (HSCs) (also referred to as Ito cells, lipocyte, perisinusoidal or fat-storing cells), and constitutes the framework of the acinus [6,12,13].

The composition of the ECM within the liver is not homogeneous. The connective tissue of the space of Disse is different from the connective tissue of the rest of the liver, such as the external capsule, septa, periductal and perivascular areas, and portal tracts [12]. Thus, two types of ECM are present in the normal liver acinus, namely the ECM sinusoidal tract in the space of Disse with HSCs, and the ECM in the portal and central vein tracts, where myofibroblasts are present [12,14,15].

Therefore, both the space of Disse and portal-central vein spaces, are considered to be interstitial spaces of the liver acinus, with the functional ability to synthesize and to degrade the ECM [12,15]. Thus, the ECM is not only a scaffold, having a mechanical role in supporting and maintaining tissue structures, but is also a complex and dynamic meshwork influencing many biological cell functions. The ECM has profound influences on the structure, viability and function of cells. However, it has also been recognized that the effect of the ECM on cells extends to immune and inflammatory cells [16]. Since the biliary microcirculation moves immersed in the ECM of the liver interstitial spaces, Disse and portal spaces, it is not bold to propose that in obstructive cholestasis the liver ECM plays a key etiopathogenic role.

## Surgical experimental cholestasis

Obstructive jaundice causes a high rate of morbidity and mortality in the human clinical field [17]. The serious repercussions of cholestasis on the liver and on the systemic level [1-3,17] have led to the creation of many experimental models so as to better understand its pathogenesis, prophylaxis, and treatment.

Several surgical techniques for developing extrahepatic cholestasis have been described, especially in the rat, based on the section of the bile duct between ligatures [18,19]. These techniques are models of reversible obstructive jaundice, since they imply a high incidence of recanalization of the extrahepatic biliary route, which can be avoided by placing the duodenum and the distal part of the stomach between the two ligated and sectioned ends of the bile duct [19]. These macrosurgical techniques of extrahepatic cholestasis, called bile duct ligation (BDL), caused development of infected hilar biliary pseudocysts by dilation of the proximal end of the bile duct. As a result, the animals died during the first 2 weeks of the postoperative period due to sepsis caused by multiple abscesses in the intraperitoneal, hepatic and pulmonary areas [20-22].

**Figure 2:**
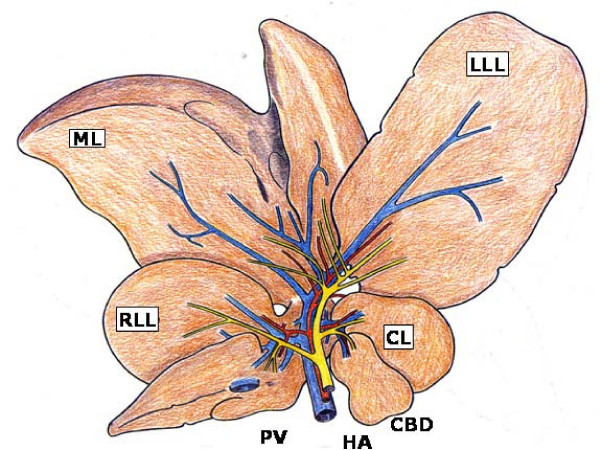
**Representation of the rat's liver made up of four lobes: median (ML), left lateral (LLL), right lateral (RLL) and caudate (CL)**. In the hilum, the relation between the portal, arterial and biliary branches is appreciated, as well as the inexistence of the gall bladder. CBD, common bile duct; HA, hepatic artery; PV, portal vein.

The hepatic parenchyma in the rat has four lobes: the right lateral, middle, left lateral and caudate lobes, which in turn have independent portal and arterial vascularization and a separate biliar drainage [23] (Figure 2).

This anatomic feature makes it possible to resect the bile ducts that drain the four lobes of the liver in continuity with the common bile duct up to the beginning of its intrapancreatic segment by means of a microsurgical technique [22,24]. First, the common bile duct is ligated and sectioned close to its intrapancreatic portion. This maneuver, which produces dilation of the extrahepatic biliary tract, facilitates the posterior dissection of the common bile duct and the lobular biliary branches of the four hepatic lobes.

**Figure 3:**
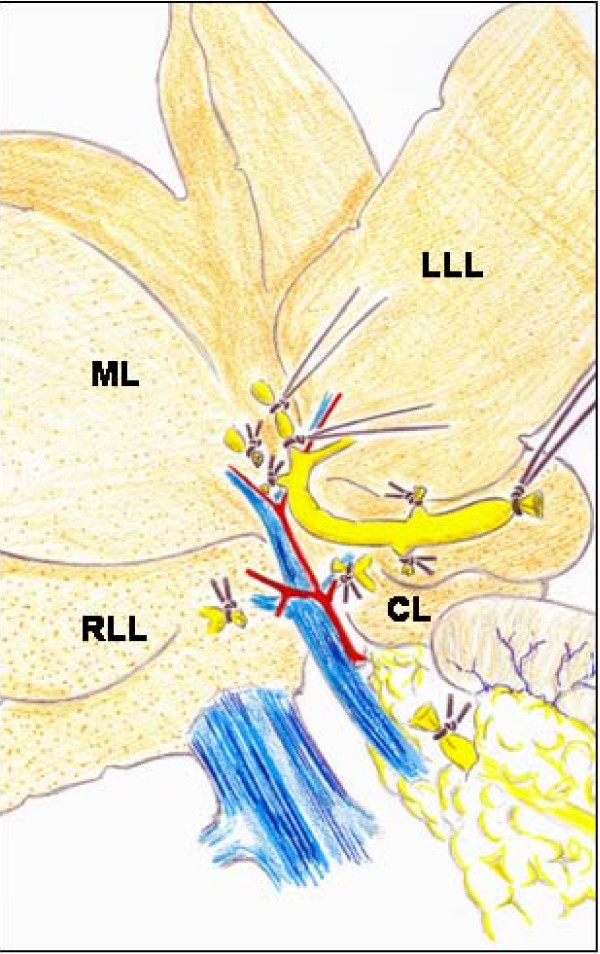
**Microsurgical technique of extrahepatic cholestasis in the rat**. The common bile duct and the lobular bile ducts are sectioned between ligations. The dissection, ligation and sectioning of the lobular bile ducts must be performed without damaging either the portal or arterial vascularization of these lobes. CL, caudate lobe; LLL, left lateral lobe; ML, middle lobe; RLL, right lateral lobe.

Once the common bile duct is sectioned, it is shifted upwards. The dissection and section between ligatures of all the biliary branches that drain the hepatic lobes is possible by using a binocular operatory microscope (Zeiss, OPMI 1-FR, Oberkochen, Germany). First, the biliary branch of the caudate lobe and then the biliary branch of the right lateral lobe are dissected, ligated and sectioned close to the hepatic parenchyma (Figure 3). The upward dissection of the extrahepatic biliary tract makes it possible to individualize, ligate and section the biliary branches draining the middle lobe and, finally, the same procedure is followed with the biliary branch of the left lateral lobe [22,24] (Figure 3).

An advantage of the microsurgical technique of extrahepatic cholestasis in the rat is the absence of large biliary pseudocyst formation, which would explain why early mortality is not present. It is possible that the absence of the hilus pseudocyst in this microsurgical model of cholestasis decreases the incidence of hepatopulmonary infection and thus prevents mortality related to sepsis [22,24].

**Figure 4:**
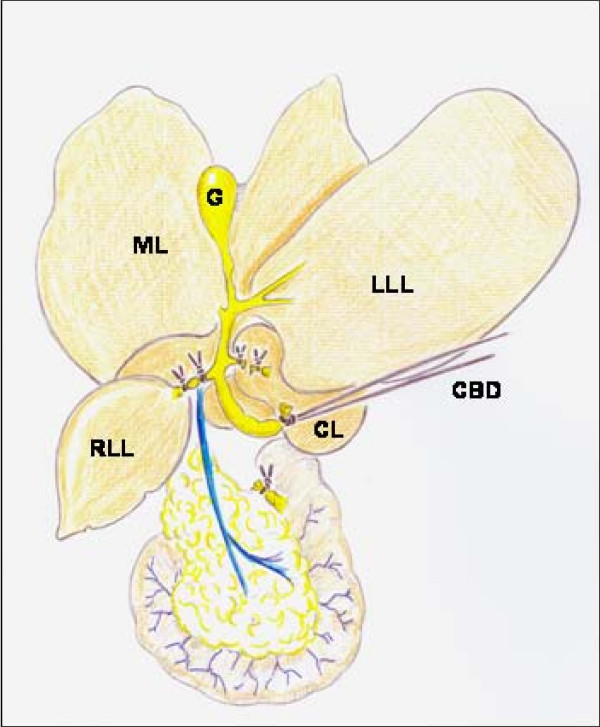
**Microsurgical technique for producing extrahepatic cholestasis in mice**. CBD, common bile duct with a long ligature to facilitate handling; CL, caudate lobe; D, duodenum; G, gall bladder; LLL, left lateral lobe; LM, medial lobe; RLL, right lateral lobe.

The macrosurgical extrahepatic cholestasis in mice consists normally in double ligation of the common bile duct with 4-0 braided silk sutures and then, sectioning between the ligatures. Finally, the cystic duct is ligated [25]. However, the microsurgical technique can be also applied to mice. The mouse liver, just like the rat's, is composed of four lobes, with the same names (Figure 4). The main difference is that the mouse liver has a gall bladder. That is why, if BDL is performed, it is followed by a marked dilation of the gall bladder, which may lead to perforation and choleperitoneum [25].

**Figure 5:**
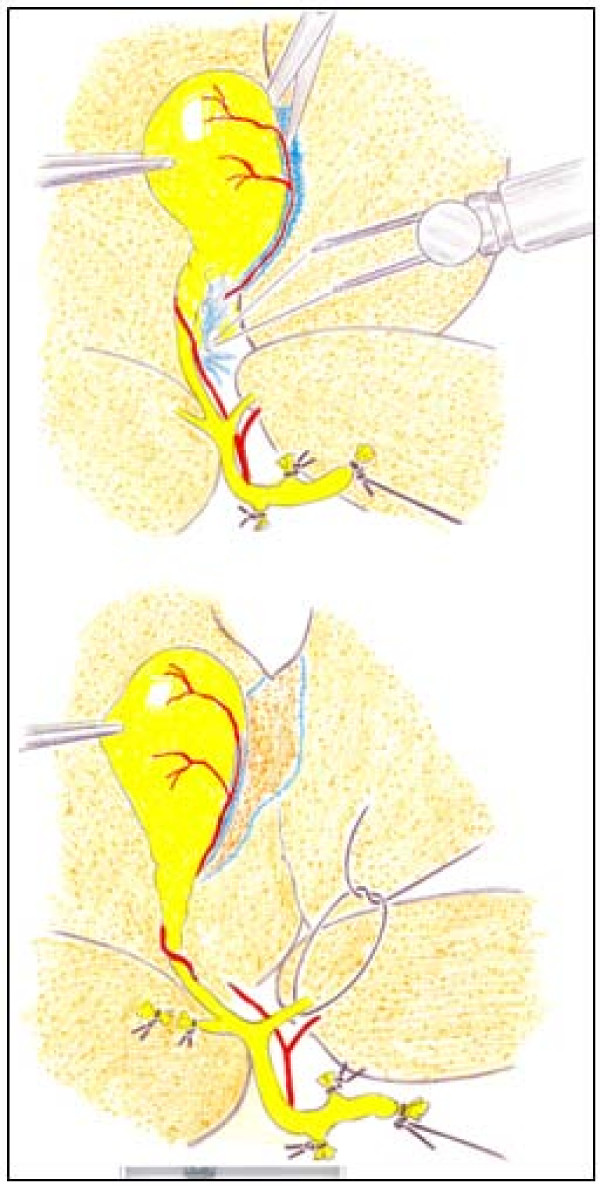
**Cholecystectomy during the production of extrahepatic cholestasis in mice**. The gall bladder is grasped with forceps near the fundus. The cystic artery is divided by caught and then the mesentery is cut with scissors (on top). The gall bladder is held in the left hand and the cystic duct is cleared of soft tissue by gentle blunt dissection. Then, the lobular bile ducts of the median and left lateral lobes (on bottom) are sectioned between ligations.

Microsurgical extrahepatic cholestasis in the mouse also consists in the resection of the four lobular bile ducts in continuity with the common bile duct (Figure 4), but cholecystectomy must be added. The dissection, ligation and section of the bile ducts from the middle and left lateral lobes are simplified if the dissection of the gall bladder and the cystic duct is performed beforehand (Figure 5).

The use of broad-spectrum prophylactic antibiotics and weekly administration of vitamin K (8 mg.kg^-1 ^intramuscularly) has been proposed to reduce the early death of the animals [25-27]. In rats with microsurgical extrahepatic cholestasis, the weekly administration of antibiotics and vitamin K makes it possible for rodents to survive over 8 weeks.

In the long-term evolution, both macrosurgical (BDL) and microsurgical experimental cholestasis models develop hepatomegaly with a marked ductular proliferation and fibrosis, but the loss of normal liver architecture, typical of cirrhosis, is seldom found [18,24,25,28]. In relation to extrahepatic alterations, jaundice, choluria [27,28], portal hypertension with enlarged spleen and collateral portosystemic circulation [24,27-30], hepatic encephalopathy [31,32] and ascytes [27] stand out. Therefore, experimental extrahepatic cholestasis is not only a good model for studying the hepatic pathology related to biliary obstruction, but also for studying extrahepatic complications.

However, the aim of this review is limited to coverage of hepatic pathology related to obstructive cholestasis. The etiopathogenic mechanisms described in its production could be compared to those that play the main role in the evolution of inflammatory response related to other injuries. [7,8,33]. It is worth mentioning that in obstructive cholestasis and other inflammatory conditions, the tissue alterations mainly occur in the interstitial space. That is why their alterations and their respective production mechanisms are mainly referred to in this context.

## The interstitial space and the inflammatory response

The interstitial space always seems to be the battlefield for inflammation, whether it is due to trauma [7,8,34], infection [8,34] or tumors [35-38].

The successive pathophysiological mechanisms that develop in the interstitial space of tissues when they undergo acute post-traumatic inflammation are considered increasingly complex trophic functional systems for using oxygen [7,8,34]. Although their extent is apparently different, the hypothetical similarity between local and systemic responses to mechanical injury could be attributed to a general response mechanism to injury in the body. This mechanism is based on the successive and predominant expression of the nervous, immune and endocrine pathological functions [7,8].

The nervous or immediate functional system has ischemia/reperfusion and edema, which work by by diffusion through the injured tissue. This trophic mechanism has a low energy requirement that does not require oxygen (ischemia), or in some circumstances the oxygen is not correctly used, with subsequent development of reactive oxygen and nitrogen species (ROS/RNS) (reperfusion) (Table 1) [7,8].

**Table 1:** Phases of the inflammatory interstitial response

**Phase**	**Response**
I. Immediate or nervous	Ischemia/reperfusion
	Edema
	Oxidative and nitrosative stress

II. Intermediate or immune	Activation of resident inflammatory cells
	Infiltration by inflammatory cells
	Toxin and bacterial translocation
	Enzymatic stress

III. Late or endocrine	Angiogenesis
	Cell proliferation
	Cell specialization
	Energetic stress

The immune or intermediate functional system produces infiltration of the injured tissue by inflammatory cells, especially by leukocytes. The immune cell residents in the interstitial space of the affected tissues and organs are also activated. Hence the capacity of these inflammatory cells for extracellular digestion by enzyme release (fermentation) and intracellular digestion (phagocytosis) could be associated with a hypothetical trophic capacity. Improper use of oxygen persists in this immune phase. Also during this phase the lymphatic circulation continues to play an important role [7,8,34,39] (Table 1).

During the evolution of the nervous and immune phase of the inflammatory response, the body looses its more specialized functions and structures. In this progressive deconstruction, depletion of the hydrocarbonate, lipid and protein stores occurs, as well as successive dysfunction and posterior failure, apoptosis, autophagy or necrosis of the specialized epithelium (that is, gastrointestinal, hepatic, pulmonary and renal). Although these alterations are considered a harmless consequence of the inflammatory response, they are also mechanisms through which there is a redistribution of carbohydrates, lipids and amino acids in the body. Consequently, the redistribution of metabolic resources responds to the different trophic requirements of the body as the inflammation progresses. Nevertheless, consumption of the substrate deposits and the dysfunction or failure of the specialized epithelia could also represent an accelerated process of epithelial dedifferentiation [40].

The hypothetical ability to involute or dedifferentiate could constitute an effective defense mechanism against injury since it could make retracing a well-known route possible, (that is, the prenatal specialization phase during the last or endocrine phase of the inflammatory response). This specialization would require or return to prominence of oxidative metabolism, and thus angiogenesis, in the affected epithelial organs, to create a capillary bed that would make regeneration of the specialized epithelial cells possible or for carrying out the repair through fibrosis or scarring [7,8,34,39,41] (Table 1).

## The liver interstitium in obstructive cholestasis

If we consider that the interstitial alterations produced during the inflammatory response are common to different conditions, the successive pathophysiological mechanisms that develop in the interstitial space of the tissues when they undergo acute traumatic inflammation [7,8] would also be expressed in the liver interstitium when suffering inflammation related to obstructive cholestasis. Thus, in the experimental cholestatic obstructive inflammatory liver disease, three inflammatory phenotypes would be expressed during its evolution: the ischemia/reperfusion phenotype (nervous), the leukocyte phenotype (immune), and the angiogenic phenotype (endocrine).

The hepatic interstitium shares both biochemical and structural characteristics with other interstitial spaces of the body. The molecules secreted by the cells occupying the body tissues interact to create a complex network, which constitutes the ECM. As there are many different tissues in the body, there are also different organizations of cells and matrices [42].

Phylogenetic data generated from recently completed genome sequencing projects have shown that the molecules of the ECM, especially those related to cell-matrix adhesion are 'ancient and exquisitely conserved in multicellular animals' [42,43]. In general, two classes of molecules are produced by the ECM: fibrous proteins (collagen, laminin and elastin) and glycosaminoglycans (GAGs) that can be non-sulfated (hialuronic acid) and sulfated [42]. Because of their high net negative charge, GAGs and proteoglycans play pivotal roles in biological processes, such as permeoselectivity of basement membranes, activation of chemokines and cytokines, cell-cell interactions and sequestration of growth factors [44,45]. This is the reason why their change has important implications in proinflammatory and anti-inflammatory activities [46-49].

Immediately after complete bile duct obstruction in the rat, an intense increase (60%) in biliary ductal pressure is produced [50] and this is quickly followed by pathological ECM changes [51]. By contrast, biliary decompression, by relieving mechanical stress, reverses liver lesions induced by BDL [52,53]. These experiments reflect the major importance that mechanical energy has in the etiopathogeny of liver injury in relation to biliary obstruction.

The response of the murine liver to the biliary obstructive injury implies its transcriptional reprogramming favoring the activation of genes regulating metabolism, cell proliferation and matrix remodeling in a time-restricted and sequential fashion [54]. Although there are a dominant activation of metabolic genes in all phases following BDL, from the immediate (1 day) to the later (21 days) phase, involvement of specific pathways varied according to the duration of obstruction [54]. Moreover, where some genes are upregulated, (that is, genes related to disruption of lipid metabolism and fibrosis) in the early stage of cholestasis, other genes are downregulated, (that is, genes involved in mechanisms of cell protection against the accumulation of toxic bile acids) [55].

The three inflammatory phenotypes hypothetically expressed in the murine liver interstitium during long-term cholestasis induced by BDL could help to integrate the etiopathogenic mechanisms that have been described. These inflammatory phenotypes would associate the genetic factors (up- and downregulated) with metabolic and histological alterations.

## The ischemia/reperfusion phenotype

After BDL, the liver rat suffers severe hemodynamic alterations, both portal and arterial, to which the effects of ischemia/reperfusion and oxidative stress can be attributed. The increase of vascular resistance in the liver portal system related to extrahepatic cholestasis results in portal hypertension [56,57] and liver ischemia, associated with a deficient production of inducible nitric oxide synthase (iNOS) and NO [58].

The biliary tree is nourished by the peribiliary plexus [59,60] and around the smaller ducts the plexus gets progressively simpler and thinner [59]. That is why it could be assumed that the increase in intraductal pressure with bile duct dilatation in extrahepatic cholestasis could induce compression of the peribiliary plexus and, consequently bile tract ischemia. However, it has been described that after the decrease in portal vein flow, an increase in hepatic arterial blood flow or a 'hepatic arterial buffer response' is produced [61]. Furthermore, after 2 weeks of BDL in the rat, a significant peribiliary plexus proliferation is produced that is drained by small venules in both the portal vein branches and hepatic sinusoids [59]. Ischemia/reperfusion injury has been in turn involved in the pathogenesis of intrahepatic cholestasis [62].

**Figure 6:**
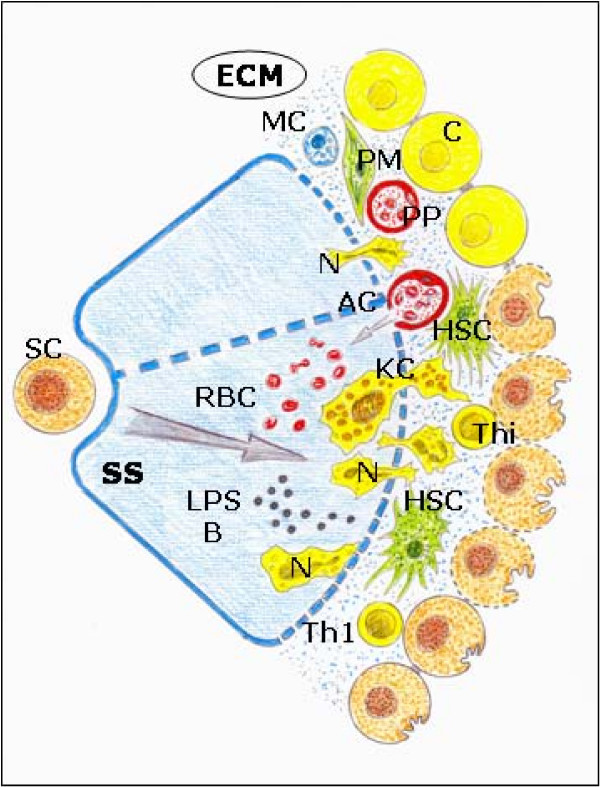
**Ischemia-revascularization and leukocytic phenotypes during the evolution of obstructive cholestasis.** Predominance of oxidative stress with edema and enzymatic stress with infiltration by leukocytes and Kupffer cell activation.  AC: arterial capillar; B: bacteria; C: cholangiocyte; ECM: extracellular matrix; HSC: hepatic stellate cell; KC: Kupffer cell; LPS: lipopolysaccharide; MC: mast cell; N: Neutrophyl; PM: portal myofibroblast; PP: peribiliary arterial plexus; RBC: red blood cells; SC: stem cells; SS: sinusoidal space; Th_1_: T cell h_1_; Thi: intraepithelial lymphocyte.

Rats subjected to BDL could have excessive accumulation of hydrophobic bile acids, which are considered the main cause of hepatotoxicity [12]. They exhibited partial impairment of mitochondrial electron transport chain functions in the liver and oxidative stress [12,27]. Retention and accumulation of hydrophobic bile salts (that is, tauro- and glicochenodeoxycholate) may decrease antioxidant activities of hepatic catalase, glutathione peroxidase, reduced glutathione (GSH) and superoxide dismutase levels and induce hepatocyte necrosis by activating mitochondrial membrane permeability transition [63-67] (Figure 6).

It is accepted that there is a strong correlation between experimental obstructive jaundice and oxidative stress [64,65,68]. However, BDL mainly impairs the liver ability of antioxidant regeneration, especially at the mitochondrial level [66]. Thus, it has been demonstrated that treatment with antioxidants improves the hepatic cellular redox status [68,69]. Indeed, antioxidants have a protective effect on hepatocellular integrity and liver functions by inhibiting reactive oxygen species formation [66,67,69].

In summary, in this early stage of BDL the insufficient supply of oxygen suffered by the liver, related to hemodynamic alterations, as well the incorrect use of oxygen derived from bile salt hepatotoxicity, would constitute the essential factors that would induce the reduction of hepatic energy metabolism. Consequently, the liver reduces its functional capacity to meet tissue metabolic needs.

Oxidative liver damage could decrease the intracellular content of proteins participating in energy production and membrane function, (that is, proteins regulating water and ion transport) [66] inducing cellular and interstitial edema. Also, increased hepatic lipid peroxidation, a high oxidative stress marker [66], can occur with increased membrane permeability, increased degradation of components of the ECM and edema [70]. The accumulation of fragments of GAG has been proposed as an important mechanism for edema formation because of the hydrophilic properties of GAGs, and particularly of hyaluronan [49,70]. GAGs attract and entrap water and ions, thereby forming hydrated gels, while permitting the flow of cellular nutrients [42,49,71]. Under inflammatory conditions hyaluronan is more polydisperse with a preponderance of lower-molecular forms, and favors edematous infiltration of the tissues [49] as well as the interstitial fluid flow and the tissue lymph pressure gradient [72]. Additionally, mechanical strain by bile tree dilatation related to BDL can lead from mechanotransduction to modifications in proteoglycans and GAGs remodeling the interstitium [73,74]. Matrix stiffness and the mechanical tension that results from cellular adhesion to stiff substrates are instrumental in determining the phenotype of many cells in culture [75].

Early mechanical stiffness has been described in the rat CCl_4 _model of fibrosis. This increase in liver stiffness appears to result from matrix crosslinking, and possibly other unknown variables, in addition to matrix quantity [76]. Also, early changes in mechanical stiffness of the liver could induce myofibroblast differentiation in early liver diseases [76,77].

The myofibroblast function endows activated hepatic stellate cells with the ability to behave like smooth muscle cells. Accordingly, activated stellate cells respond by contraction to vasoactive substances. Also, the subsequent constriction potentially regulates the diameter of liver vasculature and affects hepatic blood flow and pressure [78].

## The leukocyte phenotype

Acquiring an active immune phenotype through the cholestatic liver involves both parenchymal (hepatocytes and cholangiocytes) and non-parenchymal cells (sinusoidal endothelial cells, Kupffer cells and hepatic myofibroblasts); blood cells that migrate to the liver interstitium [77-81]. However, the interstitial space seems to orchestrate the inflammatory immune cell activity post BDL in the rat.

In particular, ECM fragments and their receptors exhibit important effects on inflammatory cells and therefore are considered to be clearly implicated in the evolution of the immune interstitial response [16,45,80]. Matrix metalloproteinase (MMPs) are a family of enzymes that degrade components of the ECM and are expressed in the diseased tissues that are inflamed [82], and are particularly present in cholestatic liver injury [83,84]. The enzymatic destruction of the ECM causes the immediate release of the mediators sequestered in its network [44,45]. Furthermore, some fragments of the ECM are molecules that have proinflammatory functions, which can enhance the immune response by activating innate and acquired immune responses [80]. Fragments of ECM proteins and hyaluronan have the ability to promote inflammation by binding Toll-like receptors (TLR)-4 and TLR-2 [81], by activating the transcriptional regulatory complex of nuclear factor (NF)κB/IκBα and by production of proinflammatory cytokines (that is, TNFα, interleukin (IL)1β) and chemokines that induce the activation and interstitial recruitment of leukocytes [80].

Upon activation, T cells undergo polarization with different cytokine profiles. Type 1 (Th1) produces interferon (IFN)γ and IL2 and type 2 (Th2) produces IL4, IL5, IL9, IL10 and IL13. In particular, Th2 cytokines are mostly involved in mediating allergic inflammation and chronic fibroproliferative disorders [79].

The liver tissue macrophages, or Kupffer cells, are mainly found in the periportal area of the lobule and, due to their location, could play a key role in ischemia/reperfusion injury [85]. But Kupffer cells are also involved in liver inflammation mediated by cholestasis through the release of biologically active substances that promote the immunopathogenic process [86] (Figure 6). Kupffer cells are clearly altered in biliary obstruction [86], with an increased phagocytic ability and a marked proinflammatory response to endotoxin and the lipopolysaccharide binding protein (LBP), which are both increased in extrahepatic cholestasis [85-87]. The hypersensibility to endotoxin in cholestasis is the cause of increased proinflammatory cytokine synthesis and increased lipid peroxidation [88], with a worsening of apoptosis and trigger progressing to necrosis [89]. However, depletion of Kupffer cells aggravates hepatocellular necrosis and inflammation in cholestasic mice [90]. The LPS-induced proinflammatory response is downregulated by high-density lipoproteins (HDL) that decrease the hepatic proinflammatory signals, restores eNOS activity and lowers portal pressure [91].

Neutrophils are key components of the initial inflammatory response to liver cholestatic injury [92]. In experimental extrahepatic cholestasis, neutrophil interstitial infiltration occurs in early phases, 3 days after BDL [5,93,94]. Biliary cells contribute to hepatic inflammation by producing neutrophil chemoattractants [4]. In long-term BDL, rats continue to show an important cell migration around the portal triad and the central vein, associated with a proinflammatory cytokine liver increase [95]. However, proinflammatory cytokines mediate a heterogeneous hepatocyte response to cholestatic stimuli, with selective hepatocyte down regulation in the periportal zone [96] (Figure 6).

Both cells, HSCs and myofibroblasts, present in the liver interstitium have the ability to express an immune phenotype. In particular, HSCs secrete a broad spectrum of inflammatory mediators, (that is, chemokines, MCP-1 and RANTES). PAF (platelet activation factor), IL8 and leukocyte adhesion molecules (ICAM-1, VCAM) are required for the recruitment and activation of leukocytes in the interstitium [6,13,14,97,98]. The homing of these cells to the liver interstitial space is favored by HSCs since these cells express MMPs, which enhance the degradation of the extracellular matrix [14,97,98]. Therefore, HSCs change the initial contractile phenotype to the immune phenotype. It is considered that these phenotypes are intricately related and even interdependent [97] (Figure 6).

Bacterial translocation is a complication of portal hypertension that is capable of inducing proinflammatory cytokines [99], and therefore is produced in BDL rats [99,100]. Furthermore, bacterial translocation provides a mechanism for the pathogenesis of bacterial infections in experimental cholestasis [100]. Increased production of TNFα may play an important role in the process of bacterial translocation in rats with cirrhosis and ascitis because TNFα blockade is able to downregulate it without increasing the incidence of systemic infections [101].

It has been proposed that the immune response, with expression of pro and anti-inflammatory mediators and recruitment of immune cells, may differ over the course of time of obstructive jaundice [102]. Thus, after the initial proinflammatory immune response, a regulating anti-inflammatory activity is established [80] in which T cells and mast cells could participate [103-106]. Dendritic cell differentiation in a cholestatic hepatic environment may lead to Th2 polarization and secretion of IL4 and IL10, rather than IFNγ [107]. In the presence of extrahepatic biliary obstruction, the activation of p38, c-Jun N-terminal kinase (JNK) and extracellular signal-regulated kinase (ERK) considered 'stress kinases' [108] would be produced. p38 MAP kinase in particular has been suggested to regulate IL10 synthesis through activation of Sp1 transcription factor rather than through the NFκB pathway [109]. Since IL10 expression is significantly upregulated 14 days after BDL mice [102], anti-inflammatory mediators may modulate the production of proinflammatory cytokines in long-term cholestasis, thus resulting in susceptibility to bacterial translocation and infection [102]. In this way, ECM molecules, like hyaluronan networks, might serve as scaffolds to prevent the loss of ECM components during inflammation and to sequester proinflammatory mediators. That is why a protective or 'counter-inflammatory' role has been suggested for the highly crosslinked hyaluronan [46,47].

Jaundice is also an important mediator of the liver inflammatory response in this experimental model of cholestasis. Bilirubin is produced via reduction of heme-derived biliverdin by biliverdin reductase [110]. Bilirubin has a number of new and interesting biochemical and biological properties [111]. In addition to having a protective role against oxidative stress [111], bilirubin has antiapoptotic and antimutagenic properties [112] as well as a strong role as an immune modulator [113,114]. Thus, in a mouse model of endotoxemia, a single dose of bilirubin in addition to its antioxidant effects also exerts a strong anti-inflammatory activity [114].

Cholestatic jaundice also occurs in the setting of sepsis [114]. Liver abnormalities in sepsis include cholestasis and hyperbilirubinemia. Hyperbilirubinemia particularly develops in sepsis in the setting of bacteriemia and precedes positive blood cultures in a third of all cases [115].

## The angiogenic phenotype

The late evolutive phase in the development of surgical experimental liver cholestasis or endocrine phase is characterized by the predominance of angiogenesis. Angiogenesis is defined as the growth of new vessels from preexisting ones [116].

Although the final objective of endothelial growth is to form new vessels for oxygen, substrates and blood cells (vascular phase), other functions could also be carried out, like antioxidative and anti-immune properties, before the new vessels are formed (prevascular phase) [117,118].

**Figure 7:**
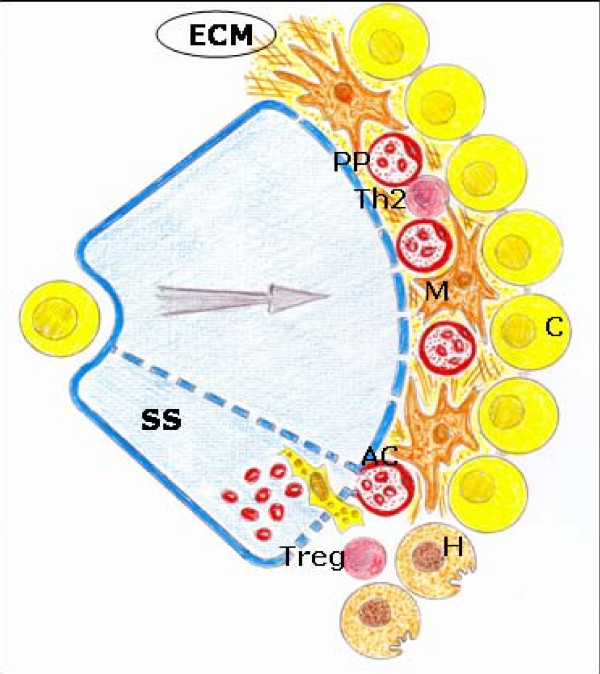
**Angiogenic phenotype during the evolution of obstructive cholestasis.** Increase in the proliferation of cholangiocytes with an important development of the peribiliar plexus and sinusoidal arterializations with hepatocytary  aplasia. AC: arterial capillar; C: cholangiocyte; ECM: extracellular matrix;H: hepatocyte; M: myofibroblast; PP: peribiliary arterial plexus; SS: sinusoidal space;  ; Th_2_: T cell h_2_; Treg: regulatory T cell.

Angiogenesis requires migration of endothelial cells into the interstitial space with the subsequent proliferation and differentiation into capillaries [118]. In BDL rats the proliferation of bile ductules, like in liver organogenesis, precedes the proliferation of the escorting microvessels. After 1 week of BDL, despite the noticeable proliferation of bile ducts, the peribiliary arterial plexus maintains its normal architecture. By contrast, after 2 and 4 weeks of BDL significant microvasculature proliferation is developed, extending from the peribiliary plexus of bile tracts [59] (Figure 7).

The main role of the cholangiocyte function in angiogenesis post BDL has been corroborated, associating hepatic artery ligation to cholestasis induced by BDL in the rat. The liver suffers increased cholangiocyte apoptosis, impaired cholangiocyte proliferation, decreased cholangiocyte vascular endothelial growth factor (VEGF) secretion and the disappearance of the peribiliary plexus. Interestingly enough, cholangiocyte functions and, thus, the integrity of the peribiliary plexus are prevented by treatment with recombinant VEGF-A [119]. Likewise, in human liver transplantation biliary regeneration occurs as an initial proliferation of the epithelial compartment, followed by the vascular compartment, which seems to be supported by induced VEGF-A expression by the epithelial compartment [120].

The ECM plays critical roles in most blood vessel formation processes. In the angiogenic process, ECM components and their fragments provide direction for regulating vessel cell migration, proliferation, differentiation and survival [42]. Integrins are the major type of ECM receptor in endothelial cells [121].

Mast cell hyperplasia is associated with the proliferation of bile ductules during extrahepatic cholestasis [105,106]. These findings suggest that mast cells accumulating in the portal triads may be involved in bile duct proliferation. At the same time, the recanalization of the ligated common bile duct led to an abrupt and transient increase in the number of mast cells associated with a rapid increase in the number of apoptotic biliary epithelial cells. These findings suggested that liver interstitial mast cells may relate to the hepatic remodeling through the induction of apoptosis [105].

**Figure 8:**
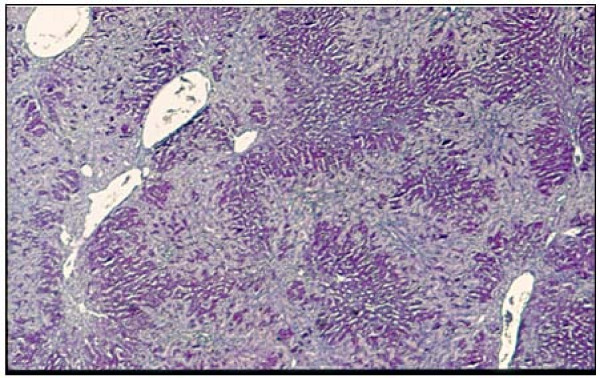
**Periportal biliary proliferation that invades Rappaport spaces I and II**. Peribiliary fibrosis is observed (hematoxylin and eosin (H&E) stain, 50 × magnification).

In long-term extrahepatic cholestasis, the predominating hepatic alteration is marked ductular proliferation with a mild portal inflammatory infiltration and apoptosis [122]. However, extrahepatic cholestasis also makes it possible to create a model of biliary fibrosis in the long term [122,123] (Figure 8). Hepatic fibrosis post BDL in rodents is the consequence of an inflammatory process of biliary origin [124,125]. Liver fibrosis is the excessive accumulation of ECM proteins including collagen [6,97,98]. A fundamental concept regarding the pathogenesis of hepatic fibrosis is that the process represents the body's wound-healing response to injury and is similar to the response of other organs to recurrent injury [97]. HSC activation is a key pathogenic feature that underlies liver fibrosis because the resulting myofibroblasts are mainly responsible for connective tissue reassembly [97,98,126]. Multiple and varied stimuli contribute to the induction and maintenance of activation, including oxidative stress, neurotransmitters (norepinephrine), the renin-angiotensin cascade, cytokines (IL4, IL13), chemokines and growth factors, that is, transforming growth factor (TGF)-β1 and connective tissue growth factor (CTGF) [13,77,97,98,122,126,127]. HSCs are further stimulated in a paracrine mode by invaded thrombocytes, polymorphonuclear leucocytes, mast cells and lymphocytes but also by activated Kupffer cells, sinusoidal endothelial cells and hepatocytes to transdifferentiate to myofibroblasts [81,98,122]. HSCs in the liver could also originate from the bone marrow and acquire the myofibroblast phenotype if the adequate, injurious microenvironment of the liver is present [13,98] (Figure 7).

Hepatic myofibroblasts are the principal cell type responsible for promoting the deposition of crosslinked fibrillar collagen in the cholestatic liver [6,98]. The coexistence of epithelial-mesenchymal transition from biliary epithelial or hepatocyte cells has also been proposed [13,15,128,129].

During the establishment of liver fibrosis, the persistence of injurious agents and the inflammatory response are followed by 'sinusoidal capillarization', which mainly consists of the transformation of fenestrated hepatic sinusoids into continuous capillaries, accompanied by the deposition of a continuous basement membrane near the endothelial cells and hepatocytes [106]. Mast cells in fibrotic livers can also be involved in hepatic arterialization [106,130]. Capillarization hinders the normal exchanges between plasma and hepatocytes and is the main cause of worsening liver function [130]. In turn, hepatic macrophages can regulate the influx of neutrophils, which may play a direct role in matrix degradation [131]. Also, infiltrating neutrophils seem to accumulate preferentially near the proliferating bile ductules and therefore they could influence the remodeling biliary epithelial cells [131].

Oxidative and enzymatic stress would, respectively, be produced during ischemia/reperfusion. Furthermore, leukocyte phenotypes in experimental obstructive cholestasis could be involved in the pathogenesis of bile duct epithelial cell proliferation and in the looping and reduplication of the duct and ductules [132]. Thus, it has been suggested that the hepatoprotective effect of honey in BDL rats could be attributed to both to their antioxidant and anti-inflammatory activities [132].

Intense biliary proliferation in the portal spaces characterizes long-term extrahepatic microsurgical cholestasis in the rat. The proliferating bile ducts invade zones 1 and 2 or Rappaport acinus, but not zone 3 or the pericentral zone [28]. This is why it could be considered an 'atypical' proliferation [11]. In essence, the pathophysiologic response of the liver, when bile flow (extrahepatic cholestasis) and/or portal venous (sinusoidal capillarization with portal hypertension) are impaired, is atrophy of the involved hepatic area and hypertrophy of the uninvolved area [133]. Histopathologically the atrophy-hypertrophy complex is characterized by septal fibrosis in the atrophic liver with biliary piecemeal necrosis, apoptosis and ductular proliferation [132,133] (Figure 7).

Cholangiocytes are considered biologically important epithelia because of the diverse array of cellular processes in which they participate, including transport of water, ions and solutes [134]. The cholangiocytes have been proposed to be the principle target cell for bile acids in the liver. Bile acids significantly alter cholangiocyte secretion, proliferation and survival [135]. Thus, bile acids can counteract the loss of bile ducts induced by cholinergic denervation in the BDL rat [136]. However, during their intense proliferation in obstructive cholestasis, proliferating cholangiocytes acquire the phenotype of neuroendocrine cells and secrete different substances including neurotransmitters (serotonin) [137], neuropeptides (opioid peptides such as met-enkephalin) [138-141], hormones (prolactin) [142] and their receptors (estrogens) [11] and growth factors, (that is, insulin-like growth factor (IGF), platelet-derived growth factor (PDGF), hepatocyte growth factor (HGF), TGFβ and VEGF) [11,136]. A great deal of evidence indicates that hepatic progenitor cell activation in the cholestatic liver is regulated by neural and neuroendocrine factors in modulating non-malignant and malignant cholangiocyte biology [136].

**Figure 9:**
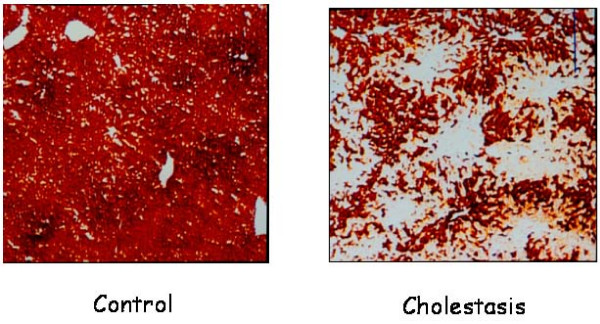
Significant inhibition of liver cytochrome oxidase activity after microsurgical extrahepatic cholestasis in the rat.

Microsurgical extrahepatic cholestasis decreases the liver cytochrome *c *oxidase activity [143]. Cytochrome oxidation accounts for more than 90% of oxygen consumption by living organisms on earth, and is essential for vital organs such as the liver [144]. Therefore, changes in the phenotype of bile ductule cells in association with a decrease in cytochrome *c *oxidase activity in the cholestatic liver may be attributable to the lower energy requirements of the neuroendocrine phenotype expressed by these cells [143] (Figure 9).

Bile duct ligation in mice has been widely used to define specific phases of acute and chronic injury and repair in the different cellular compartments of the liver [145]. Bile duct ligation elicits dynamic changes in mouse liver. Acute liver injury, with necrotic and apoptotic cell death and biliary infarcts, is followed by continuous tissue repair, lymphocyte and Kupffer cell infiltration and accumulation of collagen during the second week of postoperative evolution. In this way, Kupffer cells abrogate liver injury in mice by cytokine-dependent mechanisms that include the production of IL6 [146]. It has been demonstrated that endogenous hepatocyte growth factor (HGF) is a reasonable strategy to attenuate hepatic inflammation, necrosis and apoptosis and it has regenerative potential against cholestatic hepatitis [147]. Growth hormone (GH) administration also upregulates hepatocyte proliferation and attenuates fibrogenic response at day 28 of bile duct ligation in mice. Therefore, this endocrine pathway is a potential mechanism to modulate the liver repair response to bile duct ligation [148]. In mice biliary obstruction, as a model of liver repair response to biliary injury, many targeted genes with metabolic-, profibrotic- and proliferation-specific functions are likely involved in the acute phase of injury (first week) and in the chronic phase (4 weeks) [149]. However, although cholangiocellular proliferation occurs early in large bile ducts on days 2–3 and in small bile ducts on day 5, it only produces the rupture of normal liver architecture in the chronic evolutive stages [145]. In summary, these time-related changes in extrahepatic cholestatic mouse are similar to those previously described in rat. Since mice are frequently used in knockout studies, this experimental model could be very useful to study novel mechanistic/molecular biology insights into the pathobiology of obstructive cholestasis in rodent models.

Depression of cholangiocyte mitochondrial respiration in obstructive cholestasis could induce hypoxia-inducible factor (HIF)1α activation and overexpression, by a similar mechanism to one that has been described in tumor cells [150,151]. This supposed overactivation of HIF1α, secondary to the decrease in oxygen metabolism with reduced ATP generation, induces the obtaining of energy via other mechanisms. For example, cholangiocytes could generate sufficient reduced nicotinamide dinucleotide phosphate (NADPH) for their biological functions through the continuous replenishment of Krebs cycle intermediates [152]. By these anaplerotic mechanisms, cholangiocytes could obtain sufficient energy not only for the new functions acquired but also for proliferation [152,153].

## Conclusion

Given the plasticity of HSCs and hepatic parenchymal cells (hepatocyte-cholangiocyte axis) it should be kept in mind that while the cholestatic liver develops, they can express the same phenotypes as the post-traumatic inflammatory response [7,8] such as: an ischemic/reperfusion phenotype (hypoxic) a leukocytic phenotype (with pro- and anti-immune response) and finally an angiogenic phenotype with cholangiocyte proliferation and fibrosis.

During these evolutive phases, it could be considered that the cholagiocyte adopts a progressive metabolic complexity (neuroendocrine), which is associated with a growing structural complexity. Cholangiocytes proliferation is a key mechanism capable of conditioning the evolution of liver damage. In fact, proliferating cholangiocytes acquire the phenotype of neuroendocrine cells and secrete different substances that represent the tools of crosstalk with other hepatic cells [11]. In this way, the activation of this neuroendocrine compartment can result in the persistence of the inflammatory response, which would increase the chance for malignant cell transformation. Thus, this 'atypical' biliar proliferation seems to be able to induce an inflammatory response in the remaining liver and includes a concept of premalignancy [11].

In essence, the cholestatic liver changes include fibroblastic cells and extracellular matrix production, inflammation with an immune response, represented by lymphocytes, macrophages and dendritic cells and finally, angiogenesis, shown by newly formed blood vessels [154]. Essentially, all of the elements that constitute the inflammatory response participate in this 'host liver reaction', which may have a trophic purpose for the development of the stiffened biliary cholestatic liver. The persistence of this inflammatory response through a longer evolution would induce an 'atypical' ductular proliferation with the development of a neuroendocrine compartment [11,36-38] and, finally, a malignant cell transformation as it occurs in humans by producing biliary tract cancer (cholangiocarcinoma) [136].

## List of abbreviations

BDL: bile duct ligation; ECM: extracellular matrix; ERK: extracellular signal-regulated kinase; GAG: glycosaminoglycans; HGF: hepatocyte growth factor; HSC: hepatic stellate cell; HDL: high-density lipoprotein; HIF: hypoxia-inducible factor; IGF: insulin-like growth factor; IL: interleukin; iNOS: inducible nitric oxide synthase; IFNγ: interferon gamma; JNK: c-Jun N-terminal kinase; LBP: lipopolysaccharide binding protein; LPS: lipopolysaccharide; MMP: matrix metalloproteinase; NADPH; reduced nicotinamide dinucleotide phosphate; NO: nitric oxide; PAF: platelet activation factor; PDGF: platelet-derived growth factor; ROS: reactive oxygen species; RNS: reactive nitrogen species; TLR: Toll-like receptors; TGF: transforming growth factor; TNFα: tumor necrosis factor α; VEGF: vascular endothelial growth factor.

## Competing interests

The authors declare that they have no competing interests.

## Authors' contributions

MAA, JA and JLA conceived the integration of inflammation in experimental cholestasis. JIA, MD and JGD supported important clinical aspects and discussed the manuscript. MAA, JLA and JA wrote the final version of the manuscript.
